# Early exposure to wildfire smoke can lead to birth defects

**DOI:** 10.3389/ftox.2023.1050555

**Published:** 2023-02-24

**Authors:** Bill L. Lasley

**Affiliations:** Center for Health and the Environment, University of California, Davis, CA, United States

**Keywords:** wildfire smoke, birth defects, pregnancy, macaque monkey, wildfire, smoke exposure

## Abstract

The results of two previously published reports of the events and impacts of the *Campfire* wildfire smoke exposure that occurred in California in 2018 are amplified from the point of view of the potential toxic mechanism involved. The *Campfire* wildfire led to the exposure of a breeding colony of macaque monkeys (*Macaca mulatta*) during the peak of their breeding season in 2018–2019. Considering the timing, adverse effects, and endocrine implications reported, the cumulative evidence points to an early toxic sensitive period that can lead to birth defects in higher primates and human pregnancies. This deeper inspection of the published observations provides important caveats and useful guidance for future investigators. The unique higher primate placental–adrenal–brain axis may limit the use of many traditional toxicologic approaches. Retrospective neurological evaluations of human fetuses exposed to air pollutants during organogenesis and subsequent retrospective characterization of air samples using *in vitro* and animal models may be the best procedures to follow.

## Introduction

Recent publications (Willson et al., 2021; Capitanio et al., 2022) report evidence of a new adverse effect of wildfire smoke on a non-human primate animal model (*Macaca mulatta*). These publications expand our knowledge on wildfire smoke exposure’s adverse effects as few reports on birth defects resulting from wildfire smoke exposure have been reported. These recent publications present evidence for an *in utero* adverse effect that must occur prior to the fetal period of gestation. Reported fetal period targets of toxicity that result in deficits in growth, maturation, and time of delivery such as pre-term birth, low gestational weight, and pregnancy losses are common. The same is not true for developmental defects. Reports of wildfire smoke toxicity during the fetal period are common, whereas targets limited to the period of organogenesis have not been reported. Most of the reported toxic effects of wildfire smoke exposure on humans have been retrospective and investigated with an epidemiologic study design. This perspective highlights this new finding and clarifies how components of wildfire smoke plumes may result in birth defects in human pregnancies and how to investigate them in the future. As humans encroach further into the forested areas, more people are exposed to severe wildfire smoke for days and even weeks. Pregnant women can be exposed during an ongoing pregnancy before they may realize that they are already pregnant. Population-based studies of wildfire exposures are largely retrospective. They rely primarily on history, air monitoring records, and personal recall. Thus, the characterization of wildfire smoke exposure in early human pregnancy is a challenge, and the actual risks of wildfire smoke exposure in birth outcomes are currently ill-defined.

## Methods and materials

The timing and adverse effects of wildfire smoke were recently reported for the *Campfire* event that occurred in Northern California in 2018 (Willson et al., 2021; Capitanio et al., 2022). Hundreds of animals were exposed to that wildfire smoke plume for 2 weeks during the peak of the annual breeding season. In this perspective, an additional analysis is offered in order to provide a deeper understanding of the possible toxic mechanism(s) involved and the adverse effects of air pollution on human pregnancy.

## Results

The initial report on the *Campfire* wildfire (Willson et al., 2021) described the timing of the intense smoke exposure, its coincidence in time and space with the macaque breeding colony, and its impact on higher primate reproductive physiology during the peak of their breeding season. Based on comparisons with a 20-year archive of breeding records, no changes in the onset, duration, and/or distribution of key components of breeding/conceptions/deliveries were observed that could be associated with smoke exposure. Except for a non-statistical trend for increased pregnancy loss, there were no discernable adverse effects. The documentation of delivery dates and calculation of conception dates showed no statistically significant exposure-induced alteration in the onset of the breeding season, the length of gestations, cohort fecundity, or cohort fertility. The only noteworthy association was the preponderance of the missing pregnancies occurred only when the smoke exposure occurred in the early gestation period rather than later gestation. This is the first report to demonstrate that in a non-human primate animal, the same severe smoke exposure can have a different adverse effect on pregnancy outcomes depending on the stage of gestation in which it occurred.

Capitanio et al. (2022) focused on the evaluation of the 3-month-old neonates from the pregnancies documented in the initial descriptive report (Willson et al., 2021). Twenty years of breeding and birthing records were used to determine that some of the *Campfire* smoke-exposed neonates exhibited statistically significant deficits in both adrenal development/function and alteration in neonatal temperament. A variety of isolation and intervention stress tests were carried out on this complete cohort of neonates, and the results were compared to 20 years of similar archived test records. Tests included postures, vocalizations, and evasive behaviors. The smoke-associated adverse effects were limited to only those neonates that were exposed to smoke in the first half of gestation. At least one of the smoke-associated temperament alterations was gender-specific, which further supports the concept of an adrenal steroid disruption of neurological development.

**FIGURE 1:**
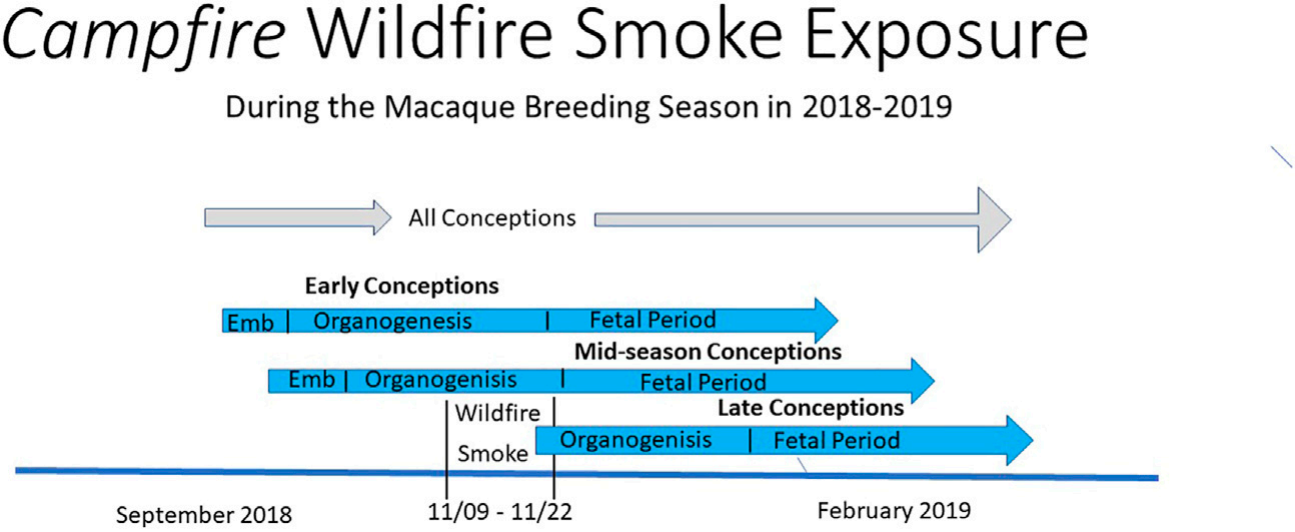
Timeline for the gestation periods: gestation periods and wildfire smoke exposure for the outdoor breeding colony during the Campfire wildfire in 2018. Affected neonates comprised the “early” and “mid” season conceptions that occurred prior to 11/22. Smoke exposure misses the period of organogenesis where those conceptions occurred in the “late” breeding season after 11/22. Organogenesis ends at the end of the first half of gestation and includes the stage of gestation when birth defects are usually induced. Emb, embryonic period.

When taken together, these two separate reports provide an argument that toxicants in wildfire smoke may include teratogenic activities that act through the placental–adrenal–brain axis in higher primate species including humans. As a result of multiple coincidences, the California *Campfire* wildfire that razed the town of Paradise in November of 2018 (Willson et al., 2021; Capitanio et al., 2022) provided new insights into the potential hazards of wildfire smoke exposure on human pregnancy. The intensity of this fire peaked in late November, and the geography and prevailing winds funneled the smoke from this fire southward toward the Sacramento Valley Delta, where it stagnated for 2 weeks. This timing coincided with the breeding season of the rhesus macaque (*Macaca mulatta*) facility that was directly in the path of the smoke plume (Willson et al., 2021), as illustrated in Figures 1–3 in Willson et al. (2021) publication.

The first and most critical coincidental factor was the timing and movement of the most severe portion of the wildfire smoke plume. This resulted in smoke and related contaminants to be funneled one hundred miles to the south, inundating the town of Davis, California, with air pollution that was documented by the California Air Resources Board to be over 40 ug/cubic meter of PM 2.5 daily for 2 weeks with a peak of 185 ug/cubic meter of PM 2.5 in late November (Willson et al., 2021). The second critical factor was the clearly defined two-week period of constant, consistent, and sustained air pollution, peaking with the peak of the breeding season for some 2,000 macaques that were housed in outdoor, open-air wire mesh enclosures (Capitanio et al., 2022). Because conceptions of individual female macaques are staggered, the intense smoky days overlapped with all stages of pregnancy in different individual animals. The intensity and quality of this exposure was similar, if not identical, in all exposed females during different but well-defined stages of fetal development (Figure 1). Therefore, an extreme two-week period of air pollution exposure occurred both day and night during different, but well-defined stages, of pregnancy. The third factor, in terms of relevance to human health, is that the macaque animal model is perhaps the best, if not the only, practical animal model for modeling human pregnancy. It was simply fortuitous that this intense wildfire smoke exposure coincided exactly with outdoor-housed macaque conceptions in late 2018.

The fourth factor was that the exposed animals were a portion of the NIH outdoor breeding colony and were maintained by the California Regional Primate Research Center (CRPRC). This facility housed hundreds of macaque monkeys being bred for research purposes. Specifically, many of the animals were permanently housed in the outdoor enclosures, and many individual animals had been the subjects of ongoing pregnancy outcome studies and, as such, comprehensive conception data had been collected for this cohort in this environment for over the previous 20 years. One of these research projects was to test and comprehensively characterize the health and temperament of neonates in a subset of animals each year. Thus, an archival record of thousands of birth outcomes and neonates from deliveries in these same outdoor facilities is available from previous non-wildfire (or control) year’s birth outcomes.

The fifth factor was the ability to restrain and digitally palpate and collect blood samples to detect, verify, and stage individual pregnancies. These data, as well as the follow-up records for deliveries, failed gestations, and missing births, were recorded and archived. These records were available for many previous years and included hundreds of individuals. The addition of measurements of the circulating maternal chorionic gonadotropin for selected individuals was used to confirm the accuracy of palpation records. Sera from the 2018 pregnant/exposed cohort were banked and retrospectively used for cortisol measurements as a further evaluation of maternal physical/chemical stressors and potential adverse effects on pregnancy status and progression (Capitanio et al., 2022). Taken together, these five factors permit and provide a unique dataset for integrative evaluations of potential toxic effects of wildfire smoke exposures that may induce birth defects in humans. Ethics will prevent relevant experimentation in humans, and thus, the macaque will be the preferred animal model. However, the non-human primate animal model has serious logistic limitations. Facilities and other resources will limit the size and intensity of a controlled study large enough to be informative. The immediate future observational data on human and animal pregnancy outcomes may be the best guidance for future investigations.

## Discussion

Perhaps the “perfect storm” occurred at the CRPRC in the fall of 2018 in terms of wildfire smoke exposure of a higher primate during pregnancy. The reporting of the events that occurred at that time was accurate, and the adverse effects of that exposure were well-documented and characterized (Willson et al., 2021; Capitanio et al., 2022). However, the nature of the toxic mechanism(s) of action was not considered in those reports, and this perspective is intended to provide additional insights that will be helpful in shaping the design and execution of future studies.

## Insights gained

The constrained breeding season of the macaque animal model was an advantage in this experiment of nature, particularly when pregnancy outcomes were to be compared between similar individuals and separate stages of pregnancy. The relatively short macaque breeding season maximized the peak smoke exposure to coincide with all stages of pregnancy in different individuals. This permitted an identification of toxic sensitive periods for wildfire smoke exposure if any existed. Since the peak exposure period lasted for only 2 weeks in late November 2018 and the six-month gestation period overlapped the exposure period by more than 8 weeks (October to February), the same exposure occurred in individual pregnancies at distinctly different stages of gestation (Figure 1). Animals that conceived in late September or early October were exposed only in the latter stages of their pregnancy. In contradistinction, the animals that conceived later in the breeding season experienced the same smoke exposure as those that conceived earlier but during early pregnancy. This pattern resulted in animals at all stages of pregnancy being exposed similarly at different stages of fetal development. Similarly, the well-defined demarcation of the intense exposure in late November (Willson et al., 2021; Capitanio et al., 2022) allowed the most severe exposure of individual pregnancies to be equally well defined. Because of the distribution of the same exposure, occurring at different stages of ongoing pregnancies, the adverse effect of that exposure could be linked to different specific stages of fetal development. This single result is key for guiding future studies of wildfire smoke toxicity for several reasons.

**FIGURE 2:**
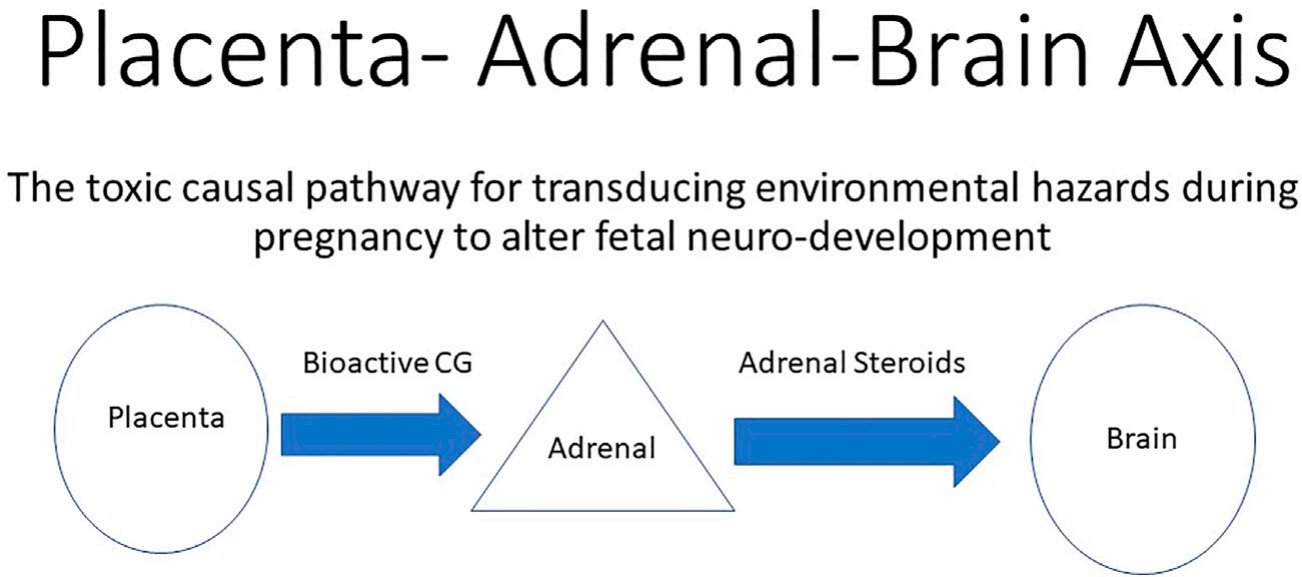
Environmental adverse effects on the placenta that directly reduces the production of bioactive chorionic gonadotropin (CG) that acts on functional LH/CG receptors in the fetal adrenals. A reduction in bioactive CG results in decreased support of the fetal adrenals. Reduced adrenal steroid hormone production leads to abnormal fetal brain development and ultimately leads to neurological defects in the neonate. In this way, adverse effects on the placenta indirectly alter neurodevelopment by altering the production of adrenal steroids.

The timing of a toxic exposure on a pregnancy defines and limits the resultant toxic mechanism(s) involved because the products of conception are constantly changing. For example, very early toxic exposures in the embryologic stage are more likely to result in pregnancy loss and not adversely affect growth (Figure 2). In contrast, exposures in mid-to late pregnancy are more likely to result in fetal growth retardation, pre-term labor, and stillbirth, as commonly reported. Only toxic exposures in gestational weeks 4–8 of the 24-week total gestational period of the macaque are likely to result in birth defects. As predicted, initial analysis of the pregnancy outcomes (Willson et al., 2021) reported a modest trend for pregnancy loss when the peak wildfire smoke exposure occurred in the first half of gestation. Capitanio et al. (2022) determined that the exposed fetuses, as 3-month-old neonates, were affected with significant differences in temperament. These adverse effects were attributed to the *Campfire* wildfire smoke exposure in the first half of gestation and largely during the period of organogenesis. This result suggests a toxic sensitive period during the gestational stage of organogenesis when teratogenic effects leading to birth defects are most likely to occur. Second, the neurological nature of the observed adverse effects provides a link to specific developmental processes in the latter first half of gestation. This timing and these events in human pregnancies are associated with the completion of critical neural links attributable, in part, to fetal adrenal steroidal control and the life-long imprinting of brain circuitry (Huang et al., 2018).

## Clues that lead to a proposed mechanism

The observation of lowered circulating cortisol in the maternal compartment during the first half of gestation (Capitanio et al., 2022, see Figure 2 in that publication) indicated that fetal adrenal steroid production was dampened rather than accelerated, as expected in response to a stressful environmental challenge. This indicates that the toxic action of the exposure was more pronounced than the stress it produced on the pregnancy. Furthermore, the expected response of the adrenals in the exposed neonates to stressors was also attenuated. Capitanio et al. (2022) also suggested suppressed adrenal development *in utero*. Since fetal adrenal growth and function are supported by placental chorionic gonadotropin (Serón-Ferré and Jaffe, 1981), this observation was consistent with the importance of the endocrine portion of the placenta and/or the fetal adrenals being targets of wildfire smoke toxicity. Earlier studies have demonstrated that some hydrocarbons can impede chorionic gonadotropin secretion for both the macaque (Chen et al., 2004) and the human (Chen et al., 2003), which permits the speculation that air-borne hydrocarbons within the wildfire smoke plume was indirectly responsible for retarded fetal adrenal development and later function (Figure 2). Since the *Campfire* smoke plume was much more than combustion of only biomass, as indicated by the rise in air-born phthalates (Willson et al., 2021), the possibility of teratogens emanating from combustion of cars, homes, and household items should be considered plausible and likely important to consider in future wildfire studies.

Macaque neonates are not well developed in the broader sense of mammalian development, but they are well-developed at birth than human neonates. Macaques have a shorter gestation (six months *versus* 9 months) than the humans, and the ability of the placenta to produce chorionic gonadotropin is limited to only 3 weeks compared to the 36 weeks in humans. Placental support of the fetal adrenals through chorionic gonadotropin is fore-shortened compared to the humans, and the timing of directing the adrenal steroid of neurologic circuitry may be compressed by 30 percent in macaques compared to that of humans. Therefore, the toxic sensitive period of fetal development may be compressed in macaques compared to humans. It would follow then that birth defects induced by wildfire smoke exposure in human pregnancies may be less severe and more difficult to detect compared to the macaque. Toxic impacts generally occur at control points in metabolism or development or where one process depends on the completion of another process. In this regard, Huang et al. (2021) reported that differences in circulating maternal bioactive androgens are associated with gender-specific differences in birth outcomes and neonatal growth trajectories. This association suggests that differences in fetal adrenal production of non-aromatizable androgens, particularly those that would escape placental aromatization in traversing the placenta, such as androstenediol, may be associated with gender-specific development of the fetal brain.

In the birth outcomes from the *Campfire* smoke exposure, the nature of the detected birth defects was predominantly neurological with an indication of gender specificity. This further incriminates fetal adrenal steroid production which plays a key role in completing the neuronal circuitry in higher centers of the brain. The gender-specific imprint of brain circuity by adrenal hormones is well documented (Konishi et al., 2019), and this life-long imprinting may begin during fetal development (Huang et al., 2021). Differences in fetal adrenal androgen production likely have gender-specific effects on the neonate. Thus, direct links are suggested between the endocrine placenta (chorionic gonadotropin), the fetal adrenals (steroids), and the brain development (neural pathways).

## A placental–adrenal–brain axis

Investigations of the adverse impact of wildfire smoke on human pregnancy bring numerous challenges to the toxicologists. While population-based studies suggest that wildfire smoke exposure is toxic to human pregnancy, few mechanisms of toxic action have been explored. Part of the reason for this lack of clarity is the nature of wildfires which are unpredictably episodic. However, the greater challenges are presented by the unique biology of human pregnancies. Most women do not recognize their own pregnancy until the fourth week of gestation and some, not until weeks later. If, as the observation of the *Campfire* exposures suggest, the sensitive period for experiencing adverse toxic effects occurs during early pregnancy, then the identification, recruitment, and sampling of control and exposed individuals during the first trimester is a particular challenge. In the case of teratogenic adverse effects, this window of toxic action is in the first 8 or 9 weeks. This time interval includes most of the first trimester of a human pregnancy.

Birth defects are some of the most devastating of all pregnancy outcomes as they impact two generations for life. They can be the result of early pregnancy toxic exposures, and unless the exposure is recognized, they are difficult to investigate. This scenario may be the case for the feto-toxic components of wildfire smoke. Although perhaps 90 percent of an ordinary wildfire smoke plume may be attributed to biomass combustion, the active toxic components may be a small contributing component that may be difficult to detect and identify. Sensitive periods for specific teratogens may be only a few days, perhaps only 2 days, and similar exposures can lead to different outcomes if the wildfire smoke composition varies on a day-to-day basis. Furthermore, human pregnancy may be unique in the role that the placenta and fetal adrenal glands act to support pregnancy in directing higher brain development (Power and Schulkin, 2006), and this may limit future experimental studies.

## Summary

A cluster of coincidental events lead to the observation of a series of associations that relate to a potential mechanism of wildfire smoke exposure toxicity. Future studies of wildfires can use these associations to develop research strategies and develop more incisive methods. The most informative associations were the timing of toxic action occurring in the first half of gestation, the induction of behavioral changes, and an indication of a gender-specific action. These associations support the nature of the toxic mechanism to be air-borne, teratogenic, and operating at a developmental control point that imprints gender-specific traits to the neonate.

These strong associations indicate that some quality of wildfire smoke can act as a teratogen in human pregnancies. This provides direction for future research endeavors. The birth defects detected in these studies were limited to alterations in temperament and indicates an ultimate neurological target of toxicity. However, suppression of cortisol secretion during early pregnancy suggests the brain is a secondary target that is downstream of suppression of fetal adrenal steroid production. Since the development, growth, and function of the fetal adrenals in higher primates are dependent on chorionic gonadotropin production (Serón-Ferré and Jaffe, 1981), a comprehensive analysis of the current information suggests the primary target of toxicity of wildfire smoke exposure may be the syncytiotrophoblast portion of the placenta. Toxicological studies of macaque pregnancies and human placental cells support this speculation as both proliferation and gonadotropin production of cultured cells are inhibited by hydrocarbons (Chen et al., 2003; Chen et al., 2004).

Unless a practical approach for recruiting and investigating early human pregnancies during wildfire exposure episodes is developed, future investigations in this research arena will need to depend on evaluation of neonates that were previously exposed to wildfire smoke in early gestation. These evaluations will need to be documented for neurological defects. Archived air samples would then be evaluated for potential teratogenic compounds and those compounds identified as candidate teratogens and tested *in vitro* with human trophoblast cells or *in vivo* using an appropriate animal model.

## Conclusion

The timing of toxic effects, the involvement of the adrenal development/function, and the nature of the neonatal neurological defects by the *Campfire* wildfire support the concept that a placental–adrenal–brain axis is a causal pathway for birth defects. The likelihood and complexity of wildfire smoke exposures will increase in the foreseeable future and continue to impact human pregnancies. The composition of air-borne components produced by these events will also become more complex and potentially more dangerous. Now that the timing and potential toxic mechanism of the most devastating of toxic actions of one wildfire has been explored, efforts to identify and safeguard against future hazards that coincide with fetal development should be a focus of the proposed new research.

## Data Availability

The original contributions presented in the study are included in the article/Supplementary Material; further inquiries can be directed to the corresponding author.
